# Case Report: Early non-convulsive status epilepticus in a preterm infant with recurrent refractory *Escherichia coli* meningitis complicated by intracranial abscess

**DOI:** 10.3389/fped.2026.1848189

**Published:** 2026-09-09

**Authors:** Jie Wu, Jing Chen, Xiaomei Xie, Fan Wang

**Affiliations:** Department of Neonatology, The Second Hospital of Lanzhou University, Lanzhou, China

**Keywords:** bacterial meningitis, intracranial abscess, non-Convulsive status epilepticus, preterm infant, surgical intervention

## Abstract

Here, we describe a male preterm infant born at 34^+5^ weeks of gestation with a birth weight of 2,390 g. At 8 days of age, he was admitted with jaundice and intermittent moaning and was diagnosed with neonatal bacterial meningitis due to *Escherichia coli*, accompanied by early non-convulsive status epilepticus (NCSE). The infant received meropenem for antimicrobial therapy and phenobarbital for seizure control. Following clinical stabilization and normalization of inflammatory markers, he was discharged. However, he subsequently developed recurrent fever and signs of disease relapse. Cerebral magnetic resonance imaging (MRI) performed during the third readmission revealed focal intracranial abscess that had not been detected on earlier imaging studies. Notably, culture of the abscess material yielded *E. coli* with an antimicrobial susceptibility profile identical to that of the initial cerebrospinal fluid isolate, confirming true relapse rather than reinfection. Surgical evacuation was performed, followed by a complete course of antibiotic therapy. The infant's symptoms resolved, and he was eventually discharged in stable condition. This case suggests that early NCSE in the acute phase of neonatal *E. coli* meningitis may be associated with a refractory, relapse-prone course.

## Introduction

Neonatal bacterial meningitis carries substantial global mortality and lifelong neurodevelopmental sequelae, with prematurity and low birth weight as leading risk factors (1, 2). More than 30% of patients show vague sepsis-like manifestations lacking meningeal signs, resulting in delayed diagnosis (3). Approximately half of survivors suffer sequelae including intracranial abscess, hydrocephalus, epilepsy and hearing loss (4–6).

Non-convulsive status epilepticus (NCSE) represents one of the most elusive atypical manifestations that produces continuous epileptiform discharges on electroencephalography (EEG) without visible convulsions, and correlates strongly with secondary brain injury, treatment failure and unfavorable neurological prognosis (7, 8). Concurrent NCSE hinders disease identification and treatment, as its occurrence indicates severe meningeal inflammation that raises risks of refractory and complicated infection.

We report a preterm infant with purulent *Escherichia coli* meningitis, whose early NCSE emerged prior to infectious relapse and intracranial abscess. This case highlights the necessity of active NCSE screening and offers reference for managing complicated neonatal meningitis.

### Patient information

A male preterm twin infant was born at 34^+5^ weeks' gestation with a birth weight of 2,390 g. Perinatal history was unremarkable. His parents were non-consanguineous, and none of his siblings or co-twin had any recent infectious illness.

On postnatal day 8, the infant was admitted to the neonatal intensive care unit (NICU) for jaundice and intermittent grunting. Physical examination identified fever, lethargy, poor feeding, hypotonia and bulging anterior fontanelle without overt clinical seizures. Routine laboratory testing revealed elevated inflammatory markers (C-reactive protein 54.03 mg/L; procalcitonin 27.00 ng/mL) and severe hyperbilirubinemia (total bilirubin 356.1 µmol/L; indirect bilirubin 313.2 µmol/L). Neonatal sepsis of bacterial origin was highly suspected, with intracranial infection and acute bilirubin encephalopathy unable to be excluded. Following blood culture collection, intravenous meropenem and phototherapy were promptly initiated. A systematic differential diagnosis was performed; acute bilirubin encephalopathy remained a possibility given the severe hyperbilirubinemia, and cerebral magnetic resonance imaging (MRI) was scheduled once the patient stabilized. Detailed history taking and cranial ultrasound ruled out hypoxic-ischemic encephalopathy, intracranial hemorrhage, and severe congenital malformations. Lumbar puncture confirmed severe purulent meningitis, with markedly elevated cerebrospinal fluid (CSF) white blood cell count (WBC; 4,021.00 × 10^6^/L, 91% polymorphonuclear cells), hypoglycorrhachia (<1.11 mmol/L) and elevated CSF protein (>3.0 g/L). Both blood and CSF cultures grew *E. coli*, susceptible to meropenem but intermediate to third-generation cephalosporins.

**Figure 1:**
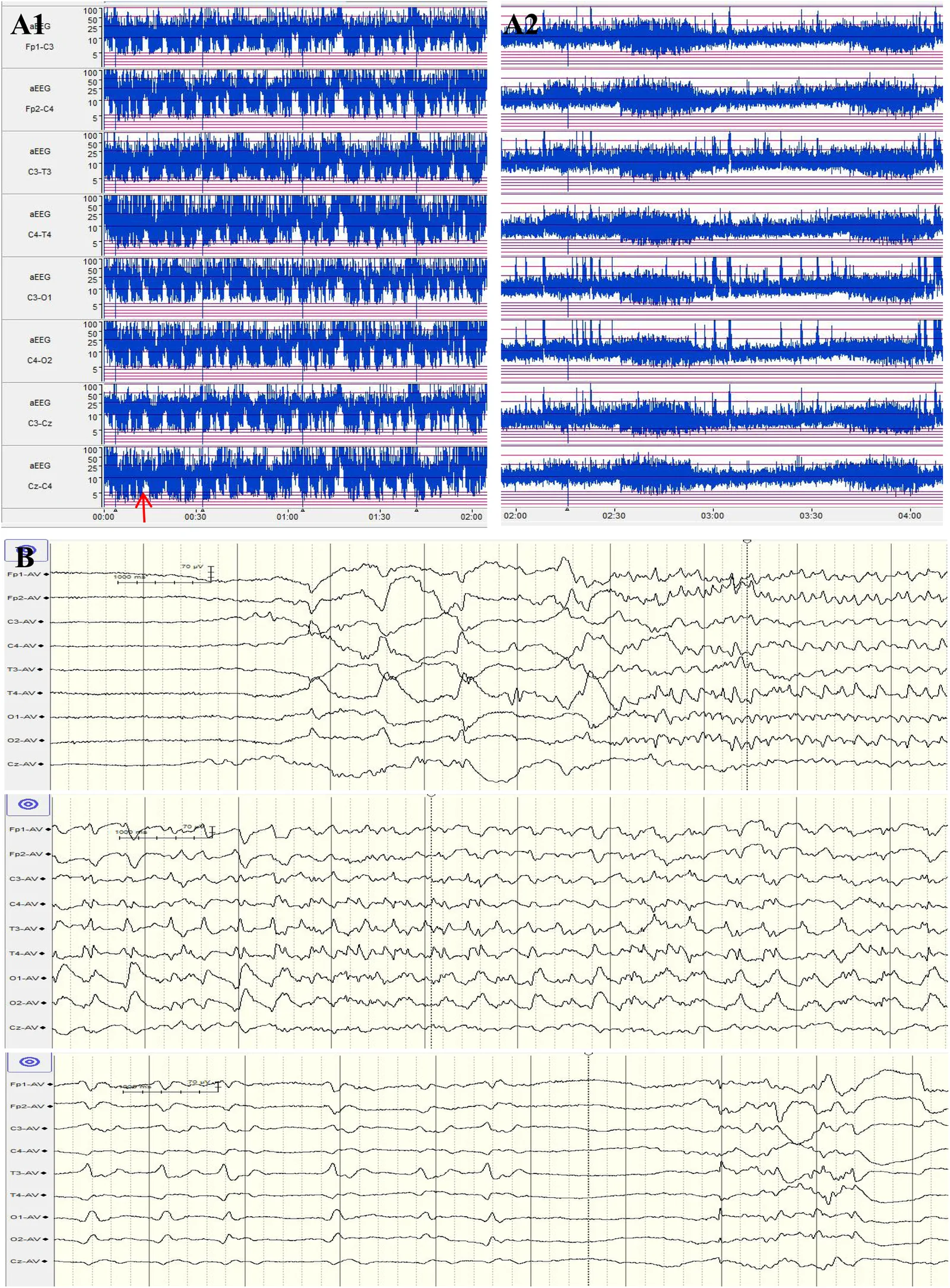
Neurophysiological monitoring findings of non-convulsive status epilepticus (NCSE) at initial admission in the preterm infant with *E. coli* meningitis. **(A)** Amplitude-integrated electroencephalography (aEEG) tracing; **(A1)** The infant's aEEG showed a discontinuous background pattern, prominent fluctuations of upper and lower trace margins, absent sleep-wake cycling, and recurrent ictal hump-like deflections (arrows) consistent with electrographic seizures. **(A2)** Following anti-seizure medication administration, aEEG exhibited continuous normal-voltage background with mature sleep-wake cycling, and no recurrent seizure-related hump-like deflections were identified. **(B)** Synchronized multichannel video-electroencephalography (vEEG) tracing. The vEEG demonstrated bilaterally symmetric, discontinuous background activity. Epileptic discharges were localized to the left frontopolar and central regions. The discharge pattern evolved progressively. Initially, there were sporadic or bursts of medium-amplitude sharp-and-slow waves, which then developed into continuous generalized medium-amplitude sharp waves intermixed with slow waves. The discharges eventually spread across all electrodes as diffuse medium-to-high amplitude sharp waves and sharp-slow wave bursts, before declining in frequency and amplitude and finally ceasing. Each electrographic seizure lasted 40–120 s, and the hourly seizure burden (SB) exceeded 20%. No associated clinical manifestations were detected on synchronized video.

Given concerns for neurological impairment in the setting of confirmed meningitis and severe hyperbilirubinemia, continuous amplitude-integrated electroencephalography (aEEG) combined with synchronized multichannel video electroencephalography (vEEG) monitoring (Nicolet EEG v32 system, Natus Neurology, USA) was initiated for cerebral function evaluation. A 9-channel neonatal montage was applied using the modified International 10–20 system (9). Monitoring was performed for at least 8 consecutive hours daily until seizure resolution, and all tracings were interpreted by an experienced pediatric neurophysiologist. The aEEG tracing showed absent sleep-wake cycling in the background with recurrent ictal hump-like deflections (Figure 1A), The corresponding vEEG exhibited progressive, high-burden epileptiform discharges originating from the left frontopolar and central regions, evolving from sporadic sharp-slow waves to generalized, high-amplitude burst discharges. Initial electrographic seizure lasted 40–120 s, with an hourly seizure burden (SB) exceeding 20%. No motor seizures were observed on synchronized video, consistent with a diagnosis of NCSE (Figure 1B). Phenobarbital was administered with a loading dose of 20 mg/kg followed by 5 mg/kg/day maintenance. Epileptiform discharges decreased by 70% within 24 h and were fully suppressed at 40 h. Follow-up EEG two weeks later showed no recurrent epileptic activity, and antiseizure medication was discontinued.

**Figure 2:**
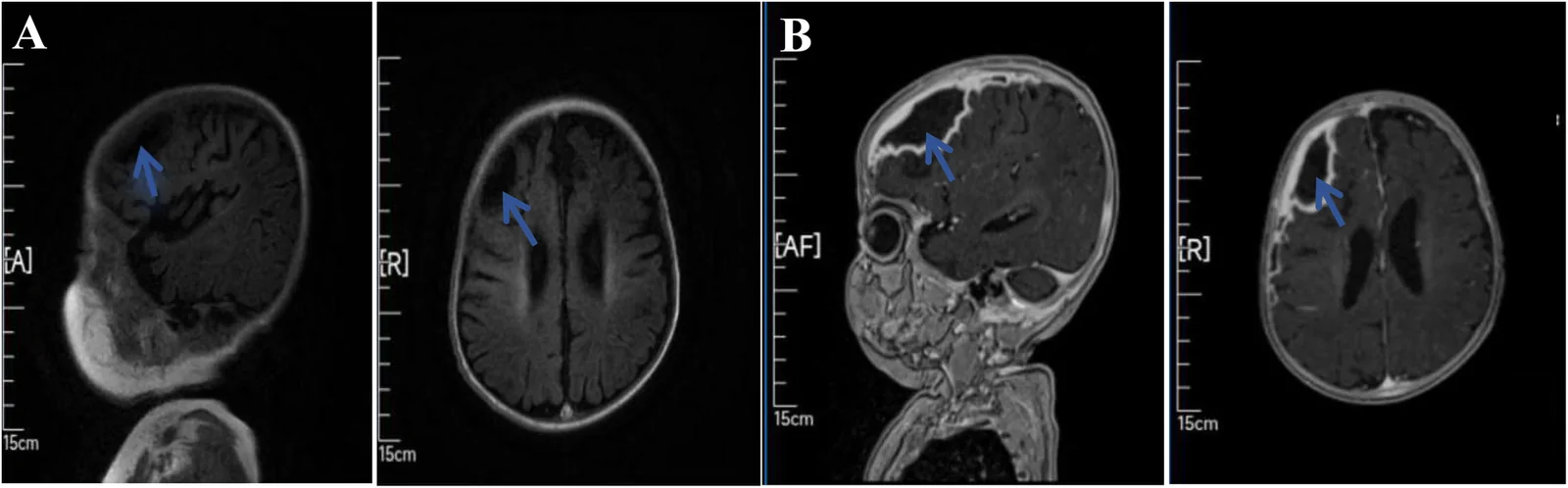
Pre-operative cerebral MRI findings in the preterm infant with *E. coli* meningitis complicated by intracranial abscess. **(A)** Pre-operative T1-weighted imaging revealed suspicion of right frontal epidural and right frontotemporal subdural abscess, with thickening of the cerebral falx. **(B)** Contrast-enhanced T1-weighted imaging acquired one week after panel **(A)** demonstrated progressive enlargement of the right frontotemporal epidural and subdural abscesses, with adjacent brain parenchyma compression and edema, thickening and enhancement of the dura mater, leptomeninges, and falx cerebri.

After a 47-day meropenem course, CSF parameters improved substantially, and the infant was discharged at postnatal day 55. However, despite initial control of NCSE, the subsequent clinical course was complicated by meningitis relapse. Five days post-discharge, the infant developed recurrent fever and lethargy; repeat lumbar puncture at an external hospital confirmed *E. coli* meningitis, and the infant received a 21-day course of meropenem. No clinical seizures were documented and no EEG monitoring was performed during this admission. Ten days after the second discharge, fever recurred again. Cerebral MRI raised suspicion of a right frontal epidural abscess with frontotemporal subdural abscess (Figure 2A). Despite one week of meropenem therapy, fever persisted, and a discrete clinical event characterized by limb tremors and transient loss of consciousness lasting approximately 1 min was observed during this period, prompting initiation of oral levetiracetam. Subsequent enhanced cerebral MRI confirmed the presence of these intracranial suppurative lesions and demonstrated their progressive enlargement (Figure 2B). After multidisciplinary discussion involving pediatric neurosurgery, radiology and clinical pharmacy teams, surgical evacuation was performed; pus culture isolated *E. coli* with antimicrobial susceptibility identical to the initial CSF isolate, confirming true disease relapse rather than reinfection. Fever resolved promptly postoperatively, and the infant completed a 51-day inpatient treatment course.

Four weeks after final discharge, cerebral MRI revealed right frontal lobe encephalomalacia as residual injury from prior intracranial abscess. Bilateral auditory brainstem response tests (ABR) showed normal hearing, but Gesell developmental assessment at corrected age 4 months 11 days confirmed moderate-to-severe neurodevelopmental delay, and long-term rehabilitation was recommended (Table 1).

**Table 1:** Timeline of the preterm infant's clinical course, diagnostic findings, and therapeutic interventions for recurrent refractory *E. coli* meningitis.

Time point	Postnatal age	Key clinical events	Key findings/Results	Interventions
1st admission	Day 8	Admitted for jaundice and grunting	Fever, lethargy; CRP/PCT↑; total bilirubin↑	Empirical meropenem; phototherapy
	Day 8–10	Intracranial infection suspected	Lumbar puncture: purulent meningitis (CS*F* testing: WBC 4,021 × 10^6^/L, 71% polymorphonuclear cells, glucose <1.11 mmol/L, total protein >3.00 g/L); blood/CSF cultures: *E. coli*	Targeted antimicrobial therapy (meropenem 40 mg/kg, q8h, iv)
	Day 8–10	initiated for cerebral function evaluation	aEEG-vEEG confirmed NCSE	Phenobarbital (loading 20 mg/kg; maintenance 5 mg/kg/day)
	Day 55	Discharged	Seizures resolved after 2 weeks of treatment; CSF improved	Discontinued antiseizure medication; Meropenem completed (47 days)
2nd admission	Day 60	First relapse	Fever, lethargy; lumbar puncture: *E. coli* meningitis	Repeat meropenem (21 days)
3rd admission	Day 96	Second relapse	Fever; CS*F* testing: WBC 1,107 × 10^6^/L, 71% polymorphonuclear cells, glucose 2.22 mmol/L, total protein 2.26 g/L; MRI: Suspected subdural abscess in the right frontotemporal region	Continued meropenem (40 mg/kg, q8h, iv)
	Day 104		Fever persisted; Clinical seizures episode	Contrast-enhanced MRI arranged; Oral levetiracetam
	Day 109	Second relapse with progression	Contrast-enhanced MRI confirmed the presence of the intracranial suppurative lesions with progressive enlargement.	Surgical evacuation of subdural abscess; Intraoperative findings: 4 cm × 2 cm × 1.5 cm subdural abscess; Pus culture: *E. coli* (susceptibility identical to initial isolate)
	Day 146	Discharged	CSF improved	Meropenem completed (50 days)
Follow-up (corrected age 4 months 11 days)	Day 174	Post-discharge neurodevelopmental assessment	MRI: right frontal encephalomalacia; ABR: normal hearing bilaterally; Gesell: moderate-to-severe developmental delay	Long-term rehabilitation recommended

CRP, C-reactive protein; PCT, procalcitonin; CSF, cerebrospinal fluid; WBC, white blood cell; aEEG, amplitude-integrated electroencephalography; vEEG, video-electroencephalography; NCSE, non-convulsive status epilepticus; MRI, magnetic resonance imaging; ABR, auditory brainstem response tests.

## Discussion

This report details the case of a premature infant with *E. coli* purulent meningitis, in whom early electrographically-detected NCSE preceded recurrent meningitis and subsequent intracranial abscess. This case suggests a potential clinical association between early-onset NCSE and a severe, complicated disease trajectory in neonatal *E. coli* meningitis, characterized by infection relapse, intracranial abscess, and adverse long-term outcomes.

Neonatal status epilepticus accounts for 15%–25% of neonatal seizure disorders, with acute symptomatic causes, including hypoxic-ischemic encephalopathy, intracranial hemorrhage, and CNS infections, comprising nearly 75% (10). Intracranial infection drives aberrant neuronal excitability, manifesting as electrographic and clinical seizures, through inflammatory blood-brain barrier disruption, direct neurotoxicity, and intense intracranial inflammation (3). NCSE is among the most easily missed critical conditions in the NICU. Unlike convulsive status epilepticus, it lacks overt motor manifestations such as visible limb convulsions, and its diagnosis relies solely on the persistent presence of epileptiform discharges on EEG (11). In the present case, although the infant suffered from severe bacterial meningitis, no clinically apparent seizures were observed throughout the entire course. This “electroclinical dissociation” is closely related to the unique neurodevelopmental characteristics of the neonatal brain. Specifically, the neonatal central nervous system is not yet fully mature, and the corticocortical and subcortical neural pathways are incompletely developed; as a result, epileptic electrical activity fails to propagate effectively to the motor cortex, and thus does not manifest as clinical seizures (12). Current clinical evidence indicates that 50%–70% of neonatal seizures detected by EEG are purely subclinical electrographic seizures, which are easily overlooked by bedside observation alone. Missed recognition and consequently delayed intervention may lead to sustained and irreversible central nervous system injury (13). Therefore, in neonates with bacterial meningitis who present with unexplained neurological depression such as lethargy, feeding difficulties, generalized hypotonia, or even apnea, a high index of suspicion for occult seizures or NCSE should be maintained, even in the absence of overt clinical convulsions.

According to the current guidelines of the International League Against Epilepsy (ILAE), vEEG is recommended as the gold standard for diagnosing neonatal seizures. However, in resource-limited settings or when continuous EEG monitoring is not feasible, aEEG can serve as an effective screening tool (14). In the present case, aEEG allowed routine NICU staff, even those without specialized neurophysiology training, to more readily identify seizure activity and initiate prompt antiseizure medication, while simultaneous continuous EEG provided precise characterization of discharge origin and burden. Moreover, post-treatment monitoring allowed for objective assessment of therapeutic response and seizure control, providing critical information for diagnostic confirmation. According to current neonatal seizure treatment guidelines, phenobarbital remains the first-line agent for neonatal seizures (15). In our patient, it reduced abnormal discharges by 70% within 24 h and achieved complete suppression by 40 h. However, substantial controversy persists over whether isolated subclinical electrographic seizures solely identified on EEG require anti-seizure medication, since antiseizure drugs may induce neuronal apoptotic damage in the immature brain (16, 17). Existing randomized controlled trials have demonstrated that administering medications to suppress subclinical discharges cannot improve short-term mortality, long-term epilepsy risk, or long-term neurodevelopmental outcomes, which does not support routine initiation of anti-seizure therapy for isolated subclinical seizures. Nevertheless, high-burden, prolonged electrographic seizures may raise cerebral metabolic demand and lead to persistent brain injury; therefore, comprehensive risk-benefit evaluation and individualized treatment are recommended in neonatal clinical management (15, 18). Of note, most published studies have centered on neonates with hypoxic-ischemic encephalopathy, and few cohorts incorporate patients with intracranial infection. In our case, given concurrent severe intracranial infection and NCSE with high SB (>20% hourly), we instituted early treatment. Similarly, domestic and international guidelines and expert consensus support pre-discharge discontinuation of anti-seizure drugs for most infants presenting with acute symptomatic neonatal seizures (15, 19). Follow-up data indicate that at 24 months corrected age, functional neurodevelopmental outcomes and long-term epilepsy risk show no meaningful differences between infants weaned off medication before discharge and those maintained on treatment, implying that prolonged anti-seizure therapy fails to improve long-term prognosis after acute seizure control (20). In our patient, follow-up EEG acquired two weeks after treatment onset showed no recurrent epileptiform discharges, hence anti-seizure agents were tapered and withdrawn.

Cumulative clinical data demonstrated that total SB exceeding 40 min is significantly correlated with elevated risks of neurodevelopmental impairments (21). Likewise, a cohort study of critically ill infants found that a maximum hourly SB threshold exceeding 20% was independently linked to unfavorable short-term neurological outcomes (22). Even after adjustment for confounders including exposure to antiseizure drugs and cerebral injury severity, high seizure burden still maintained an independent association with poorer cognitive and language performance at 18 months corrected age (23). In our patient, the early high-burden NCSE occurred in the setting of severe *E. coli* meningitis and concurrent severe hyperbilirubinemia. Given the well-established association between high SB and adverse outcomes, it is plausible that the prolonged epileptiform activity contributed to, or at least reflected, the severity of the underlying cerebral insults, with the superimposed neurotoxicity of hyperbilirubinemia further compounding the overall brain injury. The short-term developmental assessment using the Gesell Developmental Schedules identified moderate-to-severe neurodevelopmental delay, and cerebral MRI revealed right frontal lobe encephalomalacia in our patient. Studies have shown that the prevalence of post-neonatal epilepsy in patients who experienced status epilepticus during the neonatal period is as high as 46%–84% (24). In our patient, the recurrence of typical clinical seizures during the third hospitalization further corroborated this substantial long-term risk. Clinicians should fully inform caregivers of this risk, and EEG evaluation is recommended promptly if any paroxysmal events emerge during childhood.

Beyond neurodevelopmental and epileptic sequelae, the relapsing course of this case itself warrants consideration. The refractory course may be explained by biofilm formation within the subdural space, which restricts antibiotic penetration (25). In addition, the abscess cavity develops an acidic, hypoxic microenvironment that induces bacterial metabolic dormancy (26). Even if the isolate exhibits *in vitro* susceptibility to meropenem, this microenvironment may impair antibiotic tissue penetration and reduce bacterial susceptibility to standard antibiotics (27). Such latent foci may escape detection on early neuroimaging but could eventually manifest as recurrent fever and abscess formation. The refractory relapses in this infant appear to illustrate this challenging clinical scenario.

Several limitations of this case should be acknowledged. First, as a single-case study, it cannot establish a definite causal relationship between early NCSE and subsequent refractory, relapsing meningitis or intracranial abscess. This temporal observation suggests a potential clinical association, which requires further validation in larger cohorts. Second, published data show that immunodeficiency accounts for approximately 36% of recurrent meningitis cases (28). Although physiologic immune immaturity is an important host risk factor for intracranial infection in preterm infants (29), comprehensive immunological and genetic testing was not performed in the infant, thus an underlying congenital host defense defect predisposing to relapsing infection could not be definitively ruled out. Third, follow-up was relatively short, with neurodevelopmental assessment completed at a corrected age of 4 months and 11 days, limiting evaluation of long-term neurodevelopmental outcomes and subsequent epilepsy risk. These limitations should be considered when interpreting our findings.

In conclusion, this case highlights the clinical importance of early NCSE recognition in preterm infants with bacterial meningitis. The temporal observation that NCSE preceded the subsequent relapsing course and abscess formation suggests a potential association that merits attention in clinical practice. Our findings support the use of continuous EEG monitoring in neonates with severe intracranial infection and underscore the need for sustained neurodevelopmental surveillance.

### Patient perspective

The infant's parents reported that the child initially showed improvement following anti-infective and antiepileptic therapy. However, the recurrence of fever after discharge caused considerable anxiety for the family. After the diagnosis of an intracranial abscess was established, the parents expressed profound appreciation for the multidisciplinary medical care and prompt surgical treatment that ultimately led to their child's recovery. Despite the successful clinical outcome, the family remains concerned about the infant's long-term neurodevelopmental prognosis and the possibility of neurological sequelae resulting from meningitis and seizures. They have indicated their commitment to regular neurodevelopmental follow-up examinations in order to facilitate early detection and timely intervention should any developmental abnormalities arise. The parents hope that sharing this case will increase clinical awareness of the atypical presentations and potentially severe complications associated with neonatal bacterial meningitis.

## Data Availability

The original contributions presented in the study are included in the article/Supplementary Material, further inquiries can be directed to the corresponding author.

## References

[1] GBD 2023 Meningitis & Antimicrobial Resistance Collaborators. Global, regional, and national burden of meningitis, its risk factors, and aetiologies, 1990–2023: a systematic analysis for the global burden of disease study 2023. Lancet Neurol. (2026) 25(5):451–68. 10.1016/S1474-4422(26)00101-8 41911930

[2] Guarnera A Moltoni G Dellepiane F Lucignani G Rossi-Espagnet MC Campi F. Bacterial meningoencephalitis in newborns. Biomedicines. (2024) 12(11):2490. 10.3390/biomedicines12112490 39595056 PMC11591924

[3] Zainel A Mitchell H Sadarangani M. Bacterial meningitis in children: neurological complications, associated risk factors, and prevention. Microorganisms. (2021) 9(3):535. 10.3390/microorganisms9030535 33807653 PMC8001510

[4] Cheng P Qian A Zhang H Wang Y Li S Sun M. Epidemiology, microbiology and antibiotic treatment of bacterial and fungal meningitis among very preterm infants in China: a cross-sectional study. Arch Dis Child Fetal Neonatal Ed. (2025) 110(2):219–25. 10.1136/archdischild-2024-327495 39299764

[5] Peng N Fu L Liang X Lu Q. Risk factors of brain abscess in neonatal meningitis: a propensity score-matched study. Eur J Pediatr. (2023) 182(5):2215–23. 10.1007/s00431-023-04860-1 36867235

[6] Tavares T Pinho L Bonifácio Andrade E. Group B streptococcal neonatal meningitis. Clin Microbiol Rev. (2022) 35(2):e0007921. 10.1128/cmr.00079-21 35170986 PMC8849199

[7] Farmania R Garg D Sharma S. Status epilepticus in neonates and infants. Ann Indian Acad Neurol. (2020) 23(6):747–54. 10.4103/aian.AIAN_189_20 33688122 PMC7900746

[8] Willems LM Beuchat I Fisch U Sutter R Kellinghaus C Strzelczyk A. Diagnosis, treatment, and outcome prediction of non-convulsive status epilepticus in unconscious patients in intensive care units. Neurol Res Pract. (2025) 7(1):79. 10.1186/s42466-025-00435-7 41137086 PMC12553241

[9] Kuratani J Pearl PL Sullivan LR Riel-Romero RMS Cheek J Stecker MM. American Clinical neurophysiology society guideline 5: minimum technical standards for pediatric electroencephalography. Neurodiagn J. (2016) 56(4):266–75. 10.1080/21646821.2016.1245568 28436801

[10] Nagarajan L Ghosh S. Status epilepticus in the neonate. BMJ Paediatr Open. (2025) 9(1):e003202. 10.1136/bmjpo-2024-003202 40121015 PMC11931914

[11] Sutter R Kaplan PW. Electroencephalographic criteria for nonconvulsive status epilepticus: synopsis and comprehensive survey. Epilepsia. (2012) 53(Suppl 3):1–51. 10.1111/j.1528-1167.2012.03593.x 22862158

[12] Jensen FE. Neonatal seizures: an update on mechanisms and management. Clin Perinatol. (2009) 36(4):881–900. 10.1016/j.clp.2009.08.001 19944840 PMC2818833

[13] Glass HC Shellhaas RA Wusthoff CJ Chang T Abend NS Chu CJ. Contemporary profile of seizures in neonates: a prospective cohort study. J Pediatr. (2016) 174:98–103.e1. 10.1016/j.jpeds.2016.03.035 27106855 PMC4925241

[14] Pressler RM Cilio MR Mizrahi EM Moshé SL Nunes ML Plouin P. The ILAE classification of seizures and the epilepsies: modification for seizures in the neonate. Position paper by the ILAE task force on neonatal seizures. Epilepsia. (2021) 62(3):615–28. 10.1111/epi.16815 33522601

[15] Pressler RM Abend NS Auvin S Boylan G Brigo F Cilio MR. Treatment of seizures in the neonate: guidelines and consensus-based recommendations-special report from the ILAE task force on neonatal seizures. Epilepsia. (2023) 64(10):2550–70. 10.1111/epi.17745 37655702

[16] Noguchi KK Fuhler NA Wang SH Capuano S Brunner KR Larson S. Brain pathology caused in the neonatal macaque by short and prolonged exposures to anticonvulsant drugs. Neurobiol Dis. (2021) 149:105245. 10.1016/j.nbd.2020.105245 33385515 PMC7856070

[17] Kaushal S Tamer Z Opoku F Forcelli PA. Anticonvulsant drug-induced cell death in the developing white matter of the rodent brain. Epilepsia. (2016) 57(5):727–34. 10.1111/epi.13365 27012547 PMC5214662

[18] Abiramalatha T Thanigainathan S Ramaswamy VV Pressler R Brigo F Hartmann H. Anti-seizure medications for neonates with seizures. Cochrane Database Syst Rev. (2023) 10(10):CD014967. 10.1002/14651858.CD014967.pub2 37873971 PMC10594593

[19] Subspecialty Group of Neonatology, the Society of Pediatrics, Chinese Medical Association; Editorial Board, Chinese Journal of Pediatrics. Expert consensus on clinical management of neonatal seizures. Zhonghua Er Ke Za Zhi. (2022) 60(11):1127–33. 10.3760/cma.j.cn112140-20220531-00498 36319145

[20] Glass HC Soul JS Chang T Wusthoff CJ Chu CJ Massey SL. Safety of early discontinuation of antiseizure medication after acute symptomatic neonatal seizures. JAMA Neurol. (2021) 78(7):817–25. 10.1001/jamaneurol.2021.1437 34028496 PMC8145161

[21] Kharoshankaya L Stevenson NJ Livingstone V Murray DM Murphy BP Ahearne CE. Seizure burden and neurodevelopmental outcome in neonates with hypoxic-ischemic encephalopathy. Dev Med Child Neurol. (2016) 58(12):1242–8. 10.1111/dmcn.13215 27595841 PMC5214689

[22] Payne ET Zhao XY Frndova H McBain K Sharma R Hutchison JS. Seizure burden is independently associated with short term outcome in critically ill children. Brain. (2014) 137(Pt 5):1429–38. 10.1093/brain/awu042 24595203 PMC3999716

[23] Alharbi HM Pinchefsky EF Tran M-A Salazar Cerda CI Parokaran Varghese J Kamino D. Seizure burden and neurologic outcomes after neonatal encephalopathy. Neurology. (2023) 100(19):e1976–84. 10.1212/WNL.0000000000207202 36990719 PMC10186227

[24] Westergren H Finder M Marell-Hesla H Wickström R. Neurological outcomes and mortality after neonatal seizures with electroencephalographical verification. A systematic review. Eur J Paediatr Neurol. (2024) 49:45–54. 10.1016/j.ejpn.2024.02.005 38367369

[25] Hall-Stoodley L Stoodley P. Biofilm formation and dispersal and the transmission of human pathogens. Trends Microbiol. (2005) 13(1):7–10. 10.1016/j.tim.2004.11.004 15639625

[26] Coyne MJ Comstock LE. The type VI secretion system: a versatile weapon for bacterial competition and pathogenesis. Annu Rev Microbiol. (2019) 73:437–57. 10.1146/annurev-micro-020518-115550

[27] Stewart PS. Mechanisms of antibiotic resistance in bacterial biofilms. Int J Med Microbiol. (2002) 292(2):107–13. 10.1078/1438-4221-00196 12195733

[28] Masuoka S Miyazaki O Takahashi H Tsutsumi Y Hiyama T Kitamura M. Predisposing conditions for bacterial meningitis in children: what radiologists need to know. Jpn J Radiol. (2022) 40(1):1–18. 10.1007/s11604-021-01191-9 34432172 PMC8732808

[29] Melville JM Moss TJ. The immune consequences of preterm birth. Front Neurosci. (2013) 7:79. 10.3389/fnins.2013.00079 23734091 PMC3659282

